# Incidental Cone Beam CT Finding of Juvenile Pleomorphic Adenoma

**DOI:** 10.1155/2020/8862657

**Published:** 2020-11-27

**Authors:** Noura Alsufyani

**Affiliations:** ^1^Oral Medicine and Diagnostic Sciences Department, College of Dentistry, King Saud University, Riyadh, Saudi Arabia; ^2^School of Dentistry, Department of Medicine and Dentistry, University of Alberta, Edmonton, Canada

## Abstract

Pleomorphic adenoma is a benign mixed tumor composed of epithelial and myoepithelial cells, less commonly occurring in minor salivary glands or in children. A case of juvenile pleomorphic adenoma missed clinically and incidentally found in cone beam CT images is described. Clinical, radiographic, and histologic images are presented.

## 1. Introduction

Pleomorphic adenoma (PA) is a benign mixed tumor composed of epithelial and myoepithelial cells with variable morphological patterns, surrounded by fibrous capsule. Salivary gland tumors are rare; comprising 3% of all neoplasms of the head and neck region, most are benign (65-70%) [1]. PA is the most common salivary gland tumor and represents 61% of the parotid gland tumors, 18% of the submandibular, and 21% minor salivary gland tumors [1–3]. In children (16 years old and younger), benign salivary gland neoplasms are very rare (0.32-5%) with PA being the most common type [4, 5].

Pediatric PA in minor salivary glands is rare and can occur in the palate, upper lip, buccal mucosa, tongue, and gingiva [4, 5]. With small lesions, children may not be aware and adjacent bony structures are unaffected. Recurrence rate of PA in children is 2.8-46.6% depending on the type of surgical technique, and malignant transformation is higher than that in adults, 3-13%, due to long life expectancy [3–5].

There are few case reports in the dental literature on PA with radiographic examination. However, these reports are of children who initially presented complaining of an intraoral swelling. To the knowledge of the author, this is the first report of a pleomorphic adenoma in a child found incidentally in cone beam CT imaging.

## 2. Case Report

Nine-year-old girl was referred by her dentist to the Interdisciplinary Airway Clinic at the University of Alberta, Canada, for symptoms of sleep-disordered breathing and dental malocclusion. In this tertiary center, patients with maxillary-mandibular jaw disproportions with sleep-disordered breathing symptoms are evaluated by orthodontist, paediatric respirologist/sleep medicine specialist, and pediatric otolaryngology surgeon. Cone beam CT tomography of the maxillofacial complex was completed and reviewed by an oral and maxillofacial radiologist. The scan was obtained using Next Generation iCAT® (Imaging Sciences International, Hatfield, PA) with 0.3 mm voxel, 4 s of exposure, 120 kVp, and 5 mA. Acquisition of CBCT scan was based on orthodontic reasons where conventional radiography failed to provide adequate information.

### 2.1. Clinical and Radiographic Findings

#### 2.1.1. Extraoral Examination

There were no head and neck asymmetries. Palpation of the neck lymph nodes was negative for tenderness or enlargement. Temporomandibular joint examination was unremarkable.

#### 2.1.2. Intraoral Examination

The lips, tongue, labial, buccal, and vestibular mucosa were within the range of normal. Early mixed dentition with no evidence of caries, the gingiva, and alveolar mucosa are of normal color and texture. Examination of the oropharynx reveals hypertrophy of the tonsils. The examination of the soft and hard palate was initially unremarkable.

#### 2.1.3. Radiographic Examination

**Figure 1:**
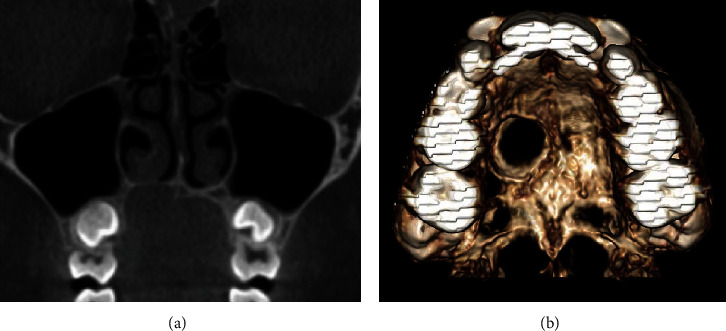
Cropped cone beam CT images of the maxilla. (a) Coronal image and (b) 3D reconstruction, showing concavity of the right hard palate, thinning, and superior displacement of floor of nasal cavity.

There were two incidental findings: first, a foreign body in the right external ear canal; second, a well-defined concavity in the right palatal process of the maxilla-premolar area (Figure 1).

In its greatest dimensions, it measured around 10 mm (width) × 10 mm (depth) × 5.7 mm (height).

**Figure 2:**
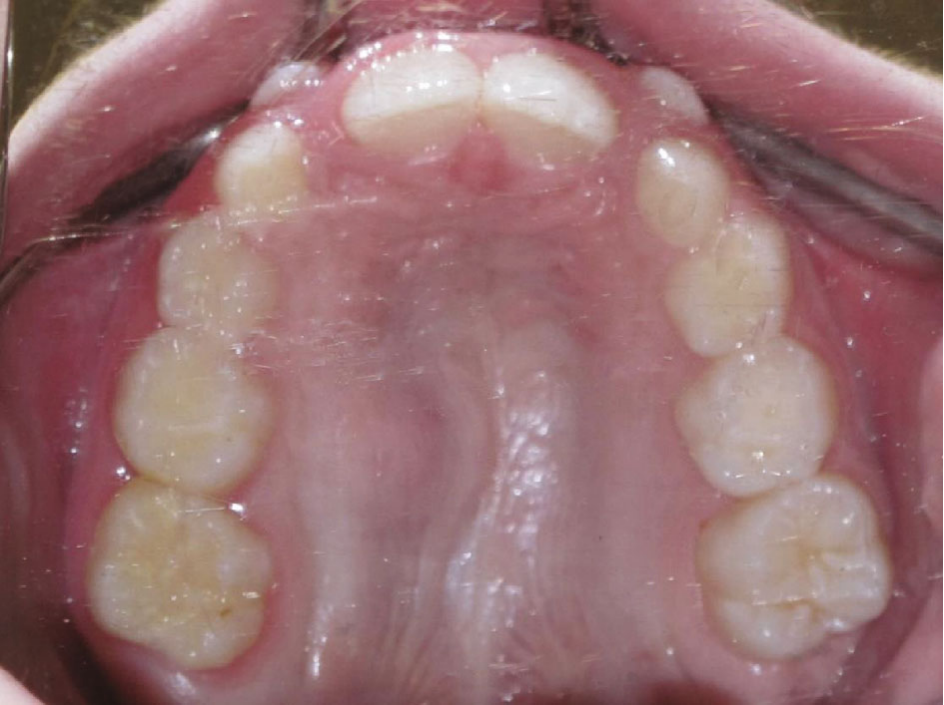
Intraoral photograph of the hard palate. Subtle swelling of the right hard palate with normal overlaying mucosa.

The concavity extended to the mid palatine suture medially and superiorly displaced the floor of nasal cavity. The soft tissue shape and density within and immediately surrounding the concavity was unremarkable, partly due to the adherent tongue-to-palate position during scan. The radiographic features were consistent with benign-cystic tumor. A careful inspection of the intraoral photographs showed mild asymmetry of the hard palate due to small swelling with normal looking mucosa (Figure 2).

### 2.2. Diagnostic Assessment

**Figure 3:**
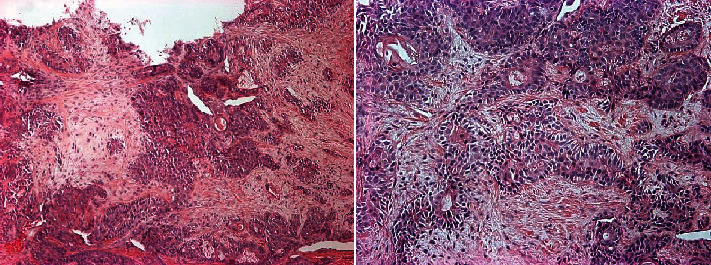
Photomicrographs of hematoxylin and eosin (H&E) staining of the pleomorphic adenoma. Ovoid or polygonal cells, duct-like structures, and fibrous stroma showing areas of myxoid changes are noted.

Punch biopsy revealed irregular sheets, trabeculae, and islands of ovoid or polygonal cells associated with duct-like structures with areas of squamoid differentiation. The supporting fibrous stroma shows zones of myxoid change (Figure 3).

Excisional biopsy specimen was negative for immunohistochemical marker S100 and confirms pleomorphic adenoma. Surface ulceration with fibrinoid necrosis and pseudoepitheliomatous hyperplasia was present.

### 2.3. Therapeutic Intervention

The patient underwent general anesthesia for the removal of adenoid hypertrophy, foreign body of the ear, and excisional biopsy of the palatal swelling. No complications were reported.

### 2.4. Follow-Up and Outcome

Four months postsurgery, the patient showed adequate healing. Eighteen months postsurgery, there were no clinical signs of recurrence.

## 3. Discussion

Pleomorphic adenoma has been reported in patients as young as 3 months up to 18 years, with slight female predilection, 1 : 1.4 [3]. Compared to adults, salivary gland tumors presenting in minor glands are higher (47.4% vs. 26.4%) and malignant lesions are higher as well (47.4% vs. 29.8%) [6]. The symptoms are similar between adults and children; nontender swelling [3, 6, 7]. PA tends to be small and fixed in minor salivary gland tumors compared to parotid where the lesion tends to be larger and mobile [3]. In this case report, the child was not aware of the palatal swelling and was subtle clinically.

Initially, the bone immediate to the soft tissue swelling would not be affected. Over time when the tumor grows enough, it may cause pressure resorption and ultimately significant erosion and perforation of the palatal bone/floor of the nasal cavity. The reported case was noted as an incidental finding in the cone beam CT due to pressure resorption of the hard palate, without break in the floor of the nasal cavity. Soft tissue changes were not evident in the cone beam CT due to lack of soft tissue resolution, and tongue position against the hard palate at the time of scan hindered the observation of a swelling silhouette. Few case reports of pediatric PA causing an erosion or displacement in the hard palate were found in the literature [8–12]. Although the radiographic examination was based on multidetector CT, the features in this cone beam CT case report are comparable.

Computed tomography and MRI are typically used in tumors of major salivary glands or large tumors of minor salivary glands. CT shows well-defined, smooth or lobulated, heterogeneous, and hypoattenuating masses compared with surrounding soft tissues and rarely presenting with mineralized foci [3]. MRI T2 or T2 short-tau inversion recovery (STIR) sequences show heterogeneous tumors with signal intensity hyperintense relative to lymph nodes, with possible hypointense rim [3].

Histologically, PA can be classified into myxoid, classic, or cellular, depending on the amount of stroma and cellular components. Myxoid type consists of myxomatous stroma, classic type is a mixture of ductal structures and myoepithelial cell in a myxomatous stroma, and cellular type has a large number of cellular elements relative to the stroma [13]. However, the morphological characteristics of cells and stroma vary widely amongst PA lesions and thus may not be straightforward.

The palate contains soft tissues other than minor salivary gland tissues. As such, fibroma, lipoma, neurofibroma, neurilemmoma, and other salivary gland tumors should also be considered in the differential diagnoses for this case. Although hematoxylin-eosin (HE) staining is the gold standard for diagnosing salivary gland tumors, immunohistochemistry (IHC) can enhance its accuracy. However, a histopathological study showed that the use of glial fibrillary acidic protein (GFAP), *α*-smooth muscle actin (SMA), CD 117, and CD 43 was not beneficial in differentiating between PA, adenoid cystic carcinoma, and polymorphous low-grade adenocarcinoma [14].

The following are markers for myoepithelial cells: vimentin and S100 but with low specificity, glial fibrillary acidic protein (GFAP) that is highly positive in PA and myoepithelioma, and p63 and CK14 that can be positive for basal and squamous epithelial cells. *α*-Smooth muscle actin (SMA), calponin, and muscle-specific actin (HHF35) are highly specific [15]. It is suggested that if SMA, calponin, and GFAP are positive, the diagnostic accuracy of PA is high [15].

Combining the clinical-radiographic presentation, histological features and negative immunological assessment (S100) for the reported case favored the diagnosis of PA, with features of myxoid and classic types.

The biological behavior of PA in young patients seems to be similar to that in adults with low chances of recurrence after adequate surgical excision [6, 7]. High recurrence rates reaching 50% are reported with inadequate surgical treatment or depending on tumor location, within 10-year follow-up [3]. Due to their longer life expectancy, recurrence and malignant transformation rates are inflated in pediatric population [3].

This report brings attention to a salivary gland tumor that was not noticed by the patient and parents or on initial oral examination. It was the bony erosion caused by the tumor that was incidentally noted upon systematic examination of the cone beam CT images. This highlights the importance of thorough clinical and radiographic examinations beyond the area of chief complaint.

Pediatric pleomorphic adenoma in the maxillofacial complex is uncommon, however should be considered in the differential diagnosis of young patients with swellings in the oral cavity. Important sites to consider are the palate, lips, tongue, and buccal mucosa. In rare occasions, diagnostic imaging could be the first line to detect such lesions incidentally by careful examination of the osseous structures and surrounding soft tissues. Consistent follow-up to monitor recurrence or malignant transformation is paramount in this young population.

## Data Availability

Data is not available. Patient consented to clinical treatment, research, and possible future publication. No consent was given to share the data.

## Additional Points

*IRB Status*. No IRB review is required for single case reports at the University of Alberta, Edmonton, Canada. The data presented in this report is not identifying.
